# The composition and functional profile of the microbial communities in human gastric cancer tissues and adjacent normal tissues: Microbial communities in gastric cancer

**DOI:** 10.3724/abbs.2021010

**Published:** 2021-12-31

**Authors:** Linli Shi, Qilin Fan, Bin Zhou, Jingjing Wu, Min Jin, Dandan Yu, Tao Zhang, Jun Song, Hongli Liu

**Affiliations:** 1 Cancer Center Union Hospital Tongji Medical College Huazhong University of Science and Technology Wuhan 430022 China; 2 Department of Gastroenterology Union Hospital Tongji Medical College Huazhong University of Science and Technology Wuhan 430022 China

**Keywords:** carcinogenesis, gastric cancer, microbial community, microenvironment, 16S rRNA

## Abstract

*Helicobacter pylori* ( *H*. *pylori*) is known to be a major risk factor for the development of gastric cancer. In recent years, increasing attention is being paid to the role of non- *H*. *pylori Helicobacters* (NHPHs) in this disease and the role of microorganisms in local tumor microenvironment. In this study, we aimed to compare the microbial community composition and the predicted functional profile in paired cancer and adjacent normal tissues of gastric cancer patients. Cancer tissues and adjacent normal tissues were collected from 10 patients with gastric cancer under endoscopy, and genomic DNA was extracted. The V3-V4 region of the 16S rRNA gene was amplified by PCR and paired-end sequencing was performed on the Illumina MiSeq System. The data was analyzed using QIIME 2 software. The results showed that microbial richness and diversity as well as genetic diversity are significantly lower in cancer tissues compared with adjacent normal tissues. At the phylum level, the dominant taxa are *Proteobacteria*, *Thermi*, *Actinobacteria*, *Bacteroidetes* and *Firmicutes*in both groups. At the genus level, some taxa, such as *Cupriavidus* and *Sphingomonas*, are significantly enriched in cancer tissues, while other taxa, such as *Ochrobactrum*, are enriched in adjacent normal tissues. Moreover, those taxa enriched in cancer tissues are associated with the synthesis and degradation of ketone bodies. In conclusion, there is a significant difference in the composition of the mucosa-related microbial communities between cancer tissues and adjacent normal tissues in patients with gastric cancer.

## Introduction

Gastric cancer is currently the fifth most prevalent malignancy and the third leading cause of cancer-associated deaths worldwide. According to statistics, there are more than 1 million new cases and approximately 780,000 deaths each year [1]. The incidence of gastric cancer varies by region and sex, with the highest rates observed in Eastern Asia and Eastern Europe, and the rates in men are on average two times more than those in women [2]. Both host and environmental factors, such as genetic susceptibility, dietary habits, lifestyles, and microbial infections, contribute to the development of gastric cancer. *H*. *pylori* infection is the strongest risk factor for gastric cancer [3]. However, only 1%–4% of *H*. *pylori*-infected individuals develop cancer, indicating that *H*. *pylori* infection alone may be insufficient to cause the disease [4].

The human digestive system is colonized with a large number of microorganisms. The stomach is a distinctive region in the digestive tract microecosystem. A unique ecological environment and a uni-que microbial community are formed in the stomach due to the secretion of gastric acid [5]. At present, research on gastric microe-cology mainly focuses on intragastric flora, which includes gastric fluid flora and gastric mucosa flora. The former is mostly from the oral cavity, while the latter can more accurately reflect the specificity of gastric flora and is more closely related to gastric mucosa-related lesions [6]. At the phylum level, the composition of the gastric flora of healthy people is similar to that in other parts of the body, which includes *Proteobacteria*, *Bacteroidetes*, *Firmicutes*, and *Actinobacteria*. Other than *H*. *pylori*, the most commonly reported genera are *Streptococcus*, *Prepotella*, *Veillonella*, *Neisseria*, *Haemophilus*, and *Fusobacterium*[ 7, 8]. However, flora of different sites in the stomach, or flora of the same site in different pathological states, can show significant heterogeneity.

Mounting evidence suggests that microorganisms play essential roles in cancer-related physiological and pathological processes, such as inflammation, immune response, tumor growth, angiogenesis, and the formation of carcinogenic metabolites and genomic instability [ 9– 15]. The molecular mechanism of *H*. *pylori* infection involved in the occurrence of gastric cancer is closely related to the chronic inflammatory response after *H*. *pylori* colonization, which leads to genomic instability, DNA damage and repair disorder, activation of oncogenic signaling pathways in normal gastric epithelial cells, and imbalanced proliferation and differentiation of stem cells. In addition, the virulence factors (CagA and VacA) and adhesion molecules of *H*. *pylori* play a promoting role in this process [ 16, 17]. Recent studies have revealed that *H*. *pylori* may interact with other bacteria in the stomach to promote the development of gastric cancer. Chronic inflammation caused by *H*. *pylori* infection can induce decreased gastric acid secretion and gastric atrophy, providing favorable conditions for harmful microorganisms and causing a microecological imbalance in the stomach [ 18, 19]. Overgrowth of bacteria has also been found in various precancerous lesions of the stomach. Taxa such as *Veillonella* and *Clostridium* can promote the synthesis of nitroso compounds, which can contribute to gastric carcinogenesis [20]. The role of NHPHs in the pathogenesis of gastric cancer has also been confirmed in mouse models [ 21, 22]. Therefore, we believe that there are other bacteria in the stomach that are related to the occurrence of gastric cancer. Currently, most studies focused on the changes in gastric microbial community composition during the progression from chronic gastritis to cancer. Our understanding of the role of NHPHs in the tumor microenvironment is not sufficient.

In this study, we investigated the differences in mucosa-associated microbial composition between cancer tissues and adjacent normal tissues in patients with gastric cancer using 16S rRNA gene sequencing. We further performed functional prediction analyses to explore the role of microbiota in the microenvironment of gastric cancer.

## Materials and Methods

### Subjects and sample collection

The inclusion criteria of patients were as follows: (1) ≥ 18 years old; (2) diagnosed of gastric adenocarcinoma via histopathology; and (3) without treatment with antibiotics, probiotics or other microecological agents during the first month preceding the study. The exclusion criteria were as follows: (1) having received antitumor treatments, including surgery, radiotherapy, chemotherapy or others; (2) having other chronic diseases of the digestive system or with primary tumors in other sites; (3) with acquired immune deficiency syndrome or autoimmune diseases; (4) in pregnancy or lactation; (5) with severe liver or renal dysfunction; (6) pica patients; and (7) with incomplete clinical data. Ten patients first diagnosed in the Gastroenterology Department of Wuhan Union Hospital between August 2020 and October 2020 were selected for the study. All patients signed the informed consent forms. Paired cancer tissues and adjacent normal tissues at least 5 cm away from the tumor were collected under endoscopy and immediately frozen and stored in a freezer at −80°C for subsequent testing. A total of 20 samples were included. This study was approved by the Ethics Committee of Tongji Medical College of Huazhong University of Science and Technology (No. 2014-041 and No. 2018-S377).

### Sample preparation and sequencing

Fresh frozen tissues were minced, ground and subject to genomic DNA extraction using the Mag-Bind soil DNA kit (Omega Bio-Tek, Norcross, USA). The quality of DNA was checked by 0.8% agarose gel electrophoresis, and DNA samples were quantified with a Nano-Drop UV spectrophotometer (Thermo Fisher Scientific, Waltham, USA). The V3-V4 region of 16S rRNA genes was amplified for each sample using the following primers: 338F, 5′-ACTCCTACGGGAGGCAGCA-3′ and 806R, 5′-GGACTACHVGGGTWTCTAAT-3′. PCR conditions were as following: 98°C for 30 s; 98°C for 15 s, 50°C for 30 s, 72°C for 30 s, 27 cycles; 72°C for 5 min, and maintained at 4°C. Amplified products were subject to 2% agarose gel electrophoresis. The target fragment was cut and then recovered using the Axygen gel recovery kit (Axygen, Shanghai, China). The purified PCR products were quantified using Quant-iT PicoGreen dsDNA Assay Kit (Thermo Fisher Scientific) and then sequenced with the Illumina MiSeq Platform (Illumina, San Diego, USA).

### Data processing and analysis

After sequencing, the raw data were converted into FASTQ format. Sequences were denoised by the DADA2 method of QIIME 2 ( https://qiime2.org). Primers were removed from the raw paired-end reads using Cutadapt ( https://pypi.python.org/pypi/cutadapt) and the sequences of unmatched primers were discarded. Sequences were then quality-trimmed, denoised, merged, and chimeras were removed. The resulting deduplicated sequences were called amplicon sequence variants (ASVs), which were clustered at 100% similarity. Then, low-abundance ASVs (ASVs whose total number of sequences was only 1 in all samples) were removed. The length distribution of the sequences contained in all samples was analyzed. Representative sequences from each ASV were aligned against the Greengenes database ( http://greengenes.secondgenome.com/) for species annotation.

The composition of the microbiota at different classification levels (phylum, class, order, family, genus, and species) in different samples was analyzed using QIIME 2. Alpha diversity was assessed using Chao1, Observed species, Shannon, Simpson, Faith’s PD and Pielou’s evenness indices. Rarefaction curves were plotted in QIIME 2 to evaluate the sequencing depth. Rank abundance curves were obtained to assess species richness and evenness using R software (R version 4.0.0, R Foundation for Statistical Computing, Vienna, Austria). The Venn diagram was drawn using R software. The beta diversity was estimated using distance matrices calculated by weighted UniFrac distances and Bray-Curtis, and was visualized using principal coordinates analysis (PCoA). Permutational multivariate analysis of variance (PERMANOVA) was then used for statistical analysis of beta diversity. Functional prediction based on the 16S rRNA gene sequence was performed with PICRUSt2 software ( https://github.com/picrust/picrust2/wiki). Differences in functional pathways between groups were analyzed using R software.

### Statistical analysis

Linear discriminant analysis (LDA) effect size (LEfSe) analysis was used to identify the taxa with significant differences between the two groups. The difference was considered significant based on LDA score>2 and *P*<0.05. The Wilcoxon signed-rank test was used to analyze the relative abundance differences between the two groups at the phylum and genus levels, and *P*<0.05 was considered statistically significant. Data analysis was performed with SPSS 24.0.

## Results

### Amplification products meet the sequencingrequirements

A total of 10 patients were enrolled in this study between August 2020 and October 2020. All patients were male, with a histologic diagnosis of gastric adenocarcinoma, and were negative for *H*. *pylori* infection. The median age at diagnosis was 65 years (range 59–76 years). The basic information of the subjects is shown in Table 1. The extracted DNAs from the patients were of good quality, and the amplification products met the sequencing requirements. A total of 735,527 high-quality sequences were obtained from these 20 samples, with an average of 36,776 sequences per sample. The length of the sequences ranged between 404 and 432 bp. Details of the sequence information are shown in Supplementary Table S1.

**Table:** **Table1** The basic information of the subjects (n=10)

Characteristics	*n* (%)
Age (years)
<65	5 (50%)
≥65	5 (50%)
Gender
Male	10 (100%)
Female	0 (0%)
Site of tumor
Gastric cardia	2 (20%)
Gastric fundus/body	1 (10%)
Gastric antrum/pylorus	7 (70%)
Histology
Adenocarcinoma	10 (100%)
Borrmann type
Borrmann 1	0 (0%)
Borrmann 2	3 (30%)
Borrmann 3	5 (50%)
Borrmann 4	2 (20%)
Lauren’s classification
Intestinal	10 (100%)
Diffuse	0 (0%)
Lymphovascular invasion
Negative	6 (60%)
Positive	4 (40%)
8th UICC/AJCC cT stage
T1	1 (10%)
T2	3 (30%)
T3	5 (50%)
T4a/4b	1 (10%)
8th UICC/AJCC cN stage
N0	3 (30%)
N1	2 (20%)
N2	4 (40%)
N3a/3b	1 (10%)
Distant metastasis
M0	8 (80%)
M1	2 (20%)
*H*. *pylori* infection
Negative	10 (100%)
Positive	0 (0%)
Family history
Yes	2 (20%)
No	8 (80%)
Drinking history
Yes	7 (70%)
No	3 (30%)
Smoking status
Smoker	6 (60%)
Nonsmoker	4 (40%)

UICC, union for international cancer control; AJCC, American joint committee on cancer.

### Microbial community diversity is significantly lower in cancer tissues

**Figure 1:**
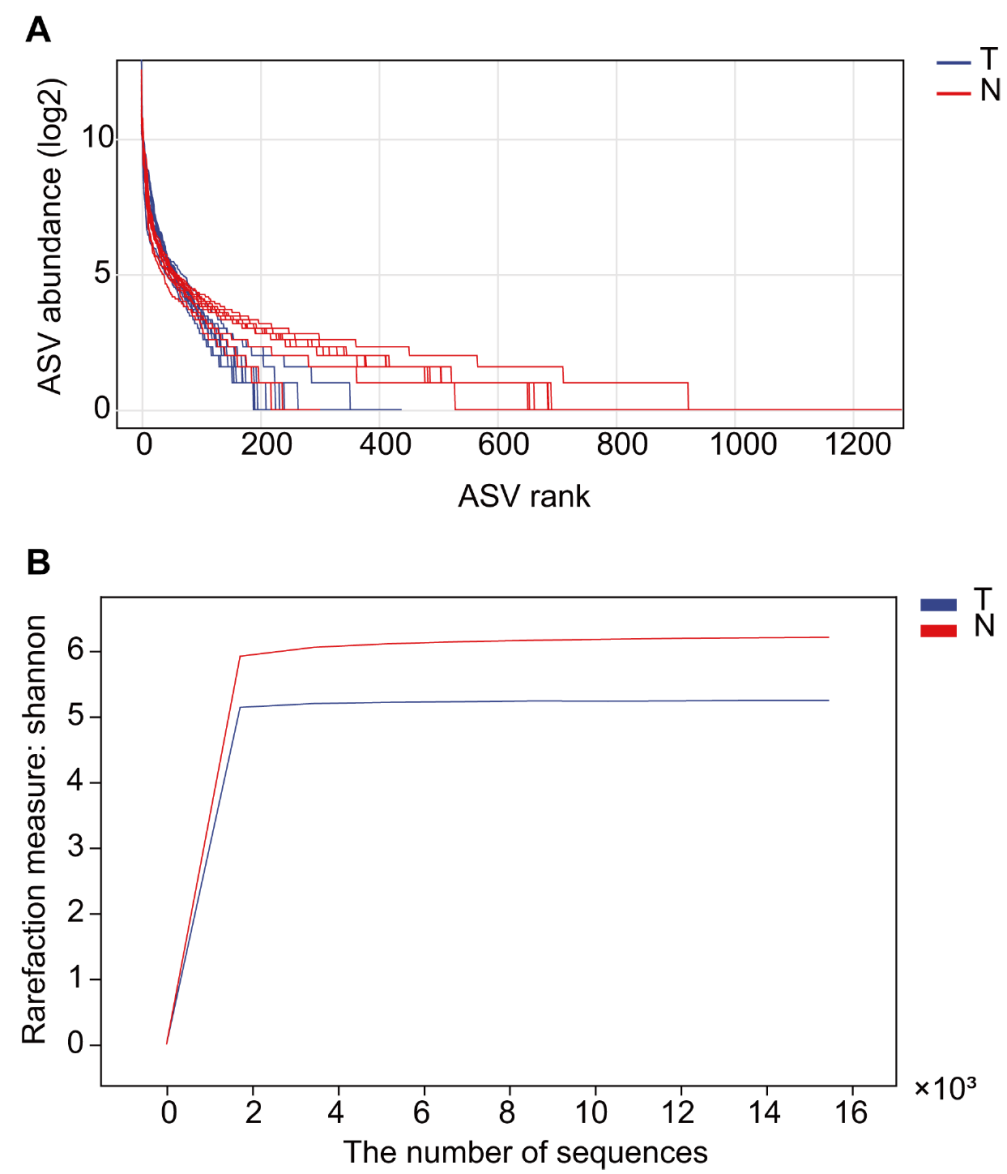
Figure1 **The rank abundance curve and the rarefaction curve**(A) The rank abundance curve. Values in ordinate were plotted on a log scale. The richness of the taxa in adjacent normal tissues was higher than that in cancer tissues. (B) The rarefaction curve. The sequencing depth was sufficient to reflect the diversity of taxa in the two groups.

**Figure 2:**
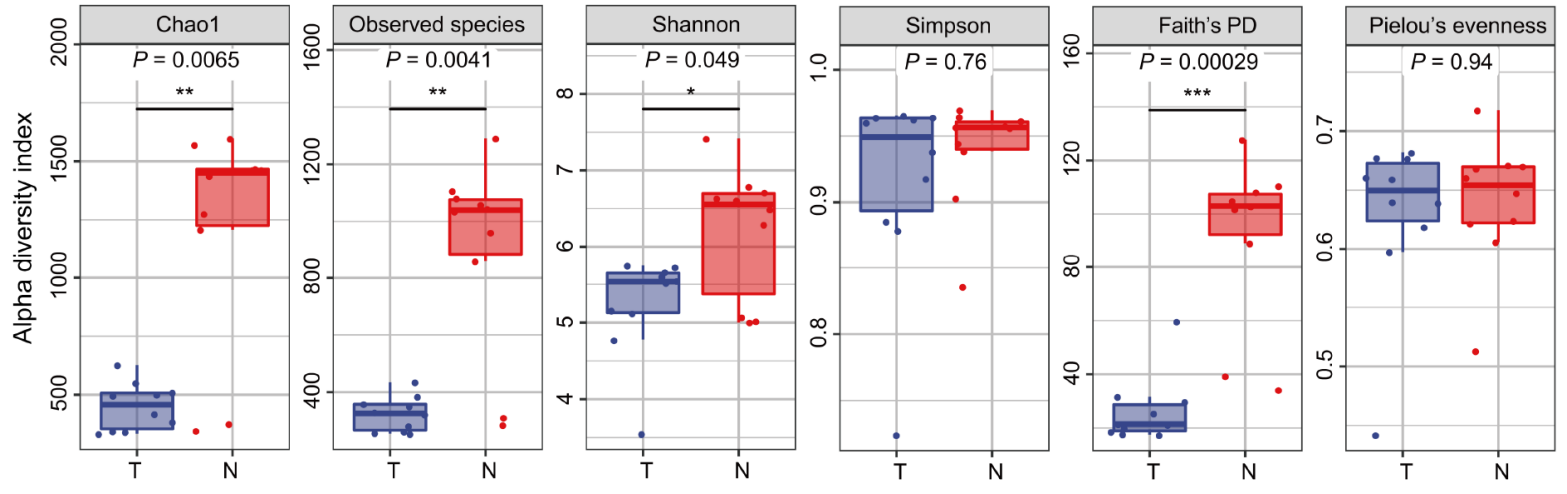
Figure2 **The alpha diversity index**The Chao1 and Observed species indices were used to reflect richness. The Shannon and Simpson indices were used to reflect diversity. Faith’s PD index was used to reflect evolutionary diversity, and Pielou’s evenness index was used to reflect evenness. *P< 0.05, **P< 0.01, ***P< 0.001.

**Figure 3:**
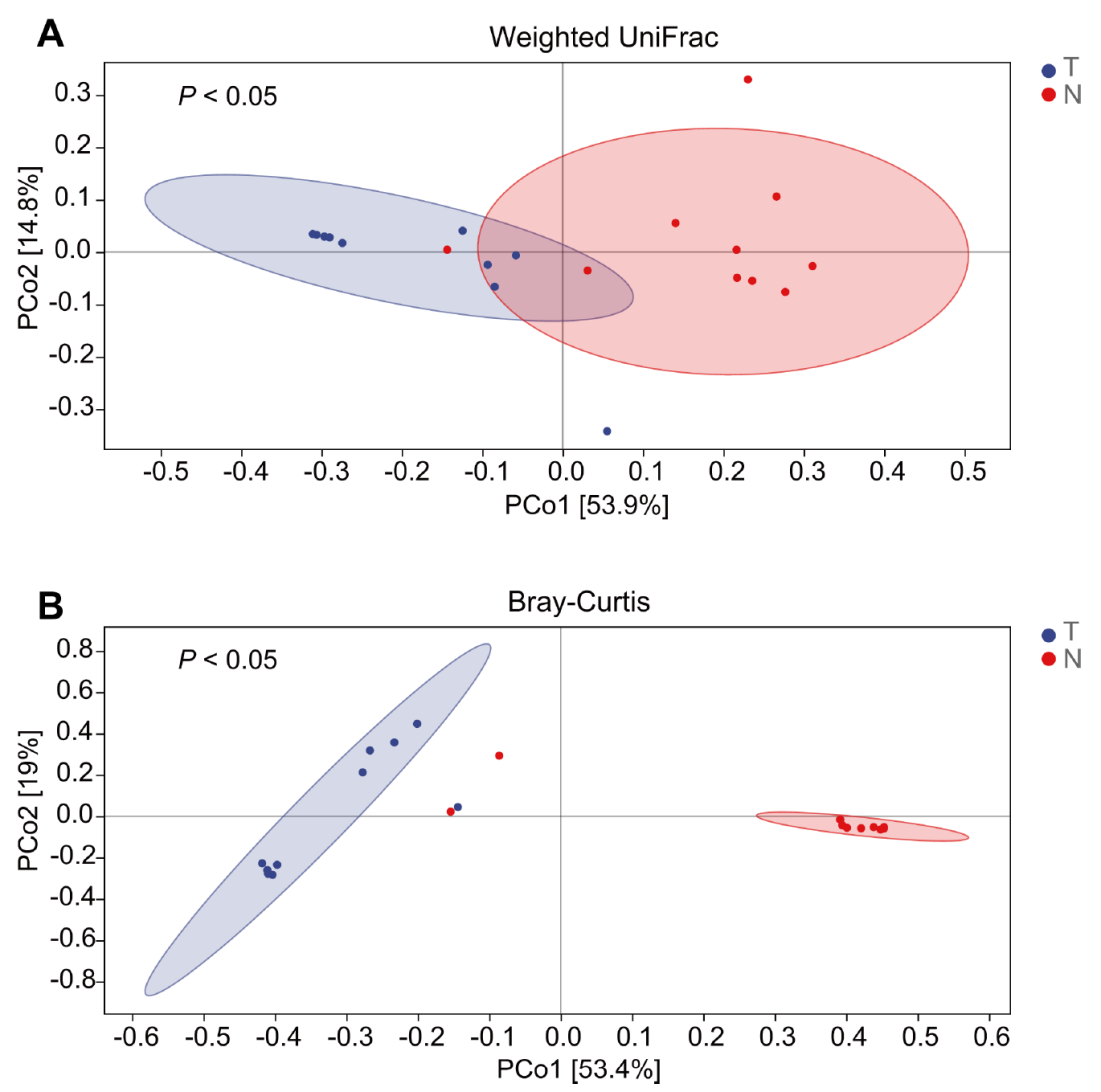
Figure3 **The PCoA analysis**Each point represents a sample. The percentages in parentheses represent the proportion of the distance matrix that the corresponding axis can explain. PERMANOVA was conducted to determine significant differences among groups.

To compare the diversity of microbial communities in cancer tissues and adjacent normal tissues, we plotted rank abundance curves and rarefaction curves. In rank abundance curves plot, each curve represents a sample, the length of the curve on the abscissa reflects the species richness, and the smoothness of the curve reflects the evenness of the distribution of species. As shown in Figure 1A, species in the adjacent normal tissues were more abundant than those in the cancer tissues. Both sets of curves were relatively steep, indicating that the species distribution was not uniform, which may be related to the existence of dominant species. The rarefaction curve can reflect the alpha diversity differences between the two groups to some extent. We plotted the curves in groups based on the Shannon index. Both sets of curves in Figure 1B had obvious plateaus, indicating that the sequencing depth was sufficient to reflect the diversity of species in the sample. The alpha diversity index analysis is shown in Figure 2. Except the Simpson index, all other indices (including Chao1, Observed species and Shannon) of cancer tissues were significantly lower than those of adjacent normal tissues ( *P*<0.05). Moreover, the genetic diversity of species in the cancer tissues was significantly reduced (Faith’s PD index: *P*< 0.001). No significant difference was observed in Pielou’s evenness index. The specific information of the alpha diversity index is shown in Table 2. To compare the differences between the two groups in microbial composition, beta diversity analysis was conducted. We performed a PCoA based on the weighted UniFrac and Bray-Curtis distance matrices. As shown in Figure 3, the microbial community of cancer tissues clustered separately from those of adjacent normal tissues ( *P*<0.05, PERMANOVA). These results indicated that there were significant differences in microbial composition between cancer tissues and adjacent normal tissues, and the diversity of microbial community was significantly lower in cancer tissues than in adjacent normal tissues.

**Table:** **Table2** Alpha diversity index of each sample

Sample ID (i)	Chao1	Observed species	Shannon	Simpson	Faith’s PD	Pielou’s evenness
T1	501.229	351.200	5.728	0.964	17.730	0.677
T2	344.640	255.200	4.777	0.879	25.464	0.598
T3	510.317	329.200	5.525	0.960	19.932	0.661
T4	496.605	434.900	5.608	0.938	59.663	0.640
T5	341.762	258.500	5.121	0.918	31.651	0.639
T6	418.343	281.300	5.547	0.963	17.432	0.682
T7	384.579	323.300	5.158	0.885	29.741	0.619
T8	550.994	360.100	5.747	0.965	18.619	0.677
T9	333.852	263.000	3.553	0.724	21.251	0.442
T10	627.164	384.300	5.661	0.964	21.524	0.659
N1	1464.310	1081.600	6.289	0.944	104.958	0.624
N2	1570.710	1107.700	6.787	0.964	102.985	0.671
N3	1208.180	858.700	5.001	0.836	89.122	0.513
N4	1275.980	961.400	6.641	0.956	110.526	0.670
N5	1438.130	1044.800	6.494	0.957	108.258	0.648
N6	1468.110	1033.800	6.614	0.958	101.835	0.660
N7	347.057	285.500	5.071	0.939	34.236	0.622
N8	1464.090	1059.700	6.716	0.962	103.057	0.668
N9	1597.430	1290.000	7.417	0.969	127.779	0.718
N10	376.163	310.900	5.016	0.903	39.359	0.606

i, order number for each sample; T, cancer tissues; N, adjacent normal tissues.

### Microbial community composition is different between cancer and adjacent normal tissues

**Figure 4:**
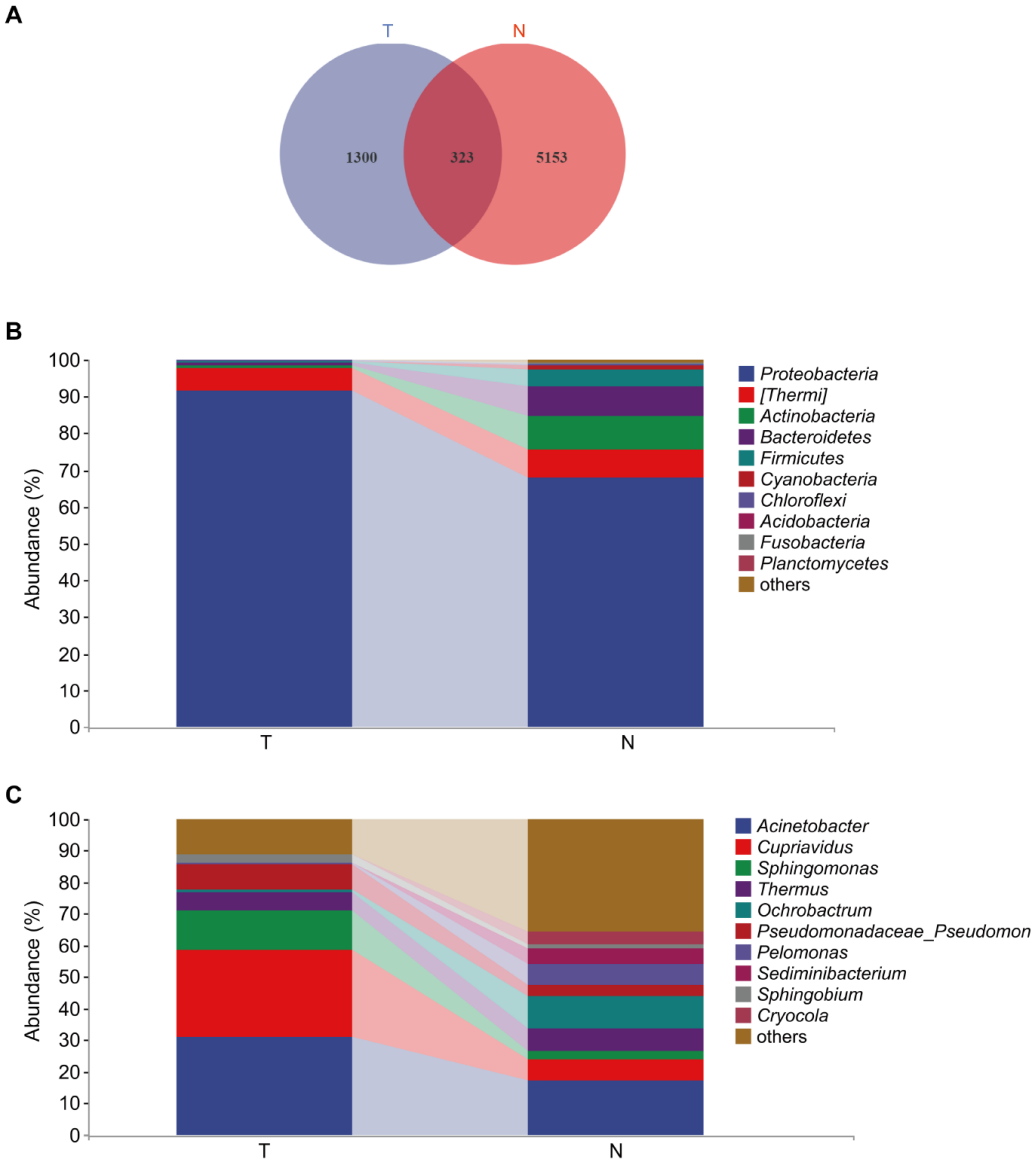
Figure4 **Venn diagram and the microbial community composition at the phylum and genus levels**(A) The Venn diagram. Each color represents a group, the overlapping regions represent the number of ASVs shared by the two groups, and the remaining two nonoverlapping parts represent ASVs unique to each group. (B) Top 10 taxa with the highest abundance at the phylum level. (C) Top 10 taxa with the highest abundance at the genus level.

To investigate the similarity and difference of ASV composition between the two groups, the Venn diagram was made using R software. As shown in Figure 4A, 1300 ASVs were unique to cancer tissues, 5153 ASVs were unique to adjacent normal tissues, and 323 ASVs were shared by both groups.

In order to study the difference of relative abundance between the two groups, we performed a taxonomic composition analysis at the phylum and genus levels, and identified 10 most abundant taxa. At the phylum level, the dominant phyla in both groups were *Proteobacteria*, **Thermi*, Actinobacteria *, *Bacteroidetes* and *Firmicutes*, accounting for more than 97% of the community ( Figure 4B), but their abundance was significantly different between the two groups. Compared with adjacent normal tissues, cancer tissues had higher abundance of *Proteobacteria* ( *P*<0.01), but lower abundance of *Actinobacteria*, *Bacteroidetes* and *Firmicutes* ( *P*<0.01). At the genus level, the main dominant genus of the two groups was *Acinetobacter* ( Figure 4C). As for the secondary dominant genera, the abundance of *Cupriavidus* ( *P*<0.05) and *Sphingomonas* ( *P*<0.01) was significantly higher in cancer tissues than in adjacent normal tissues, whereas the abundance of *Ochrobactrum*, *Pelomonas* and *Sediminibacterium* was significantly lower in cancer tissues than in adjacent normal tissues ( *P*<0.01). The abundance information of the above taxa is shown in Supplementary Tables S2 and S3.

**Figure 5:**
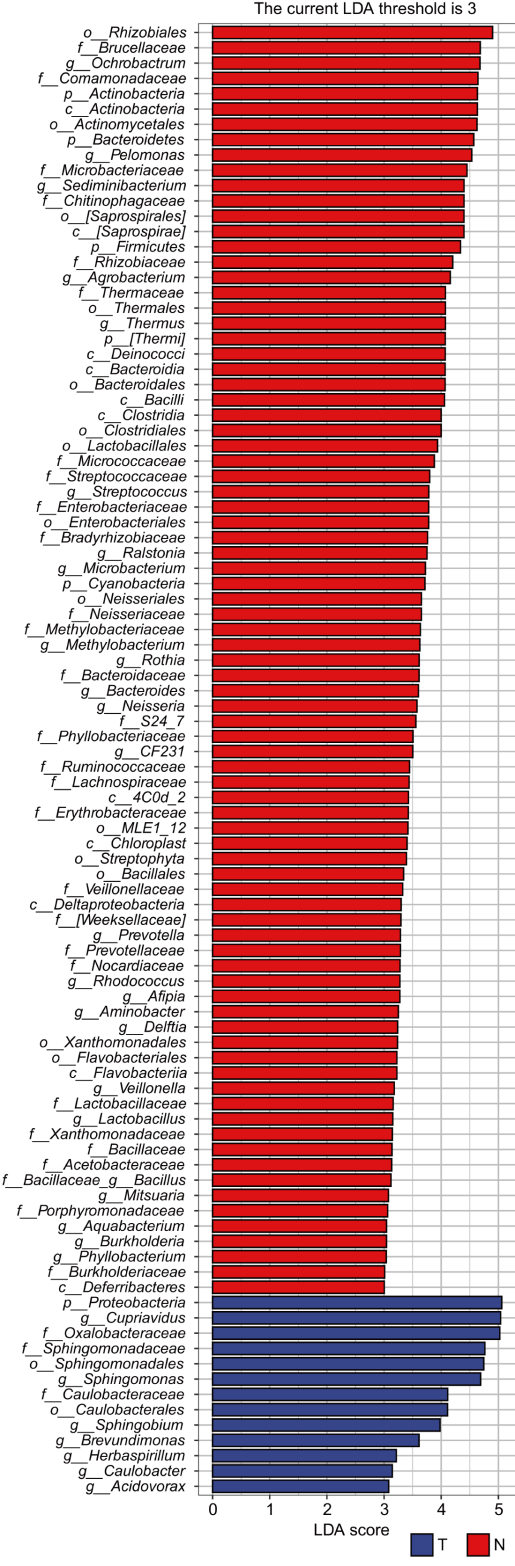
Figure5 **The LEfSe analysis**The ordinate is the taxa with significant differences between groups, and the abscissa is the logarithmic score of LDA analysis for each taxon. The longer the length, the more significant the difference in taxon abundance between groups. The color of the bar graph indicates the group with a higher relative abundance of the taxon.

To identify the taxa with statistically significant differences in abundance between the two groups and the contribution of each taxon to the differences, the LEfSe analysis was performed. As shown in Figure 5, when the threshold for the LDA score was set at 3, there were 96 taxa with significant differences in abundance, distributed at different levels of phylum, class, order, family, and genus. The larger the LDA score, the greater the influence of taxon abundance on the difference between the two groups. At the genus level, 7 taxa were enriched in cancer tissues, including *Cupriavidus*, *Sphingomonas*, *Sphingobium*, *Brevundimonas*, *Herbaspirillum*, *Caulobacter*, and *Acidovorax*, while 25 taxa were enriched in the adjacent normal tissues, including *Ochrobactrum*, *Pelomonas*, *Sediminibacterium*, *Agrobacterium*, *Thermus*, *Streptococcus*, *Ralstonia*, *Microbacterium*, *Methylobacterium*, *Rothia*, *Bacteroides*, *Neisseria*, *etc*. These results suggested that both groups have their own dominant taxa, and the abundance differences are mainly reflected at the genus level and below.

### Taxa enriched in cancer tissues are related to ketone body synthesis and degradation

**Figure 6:**
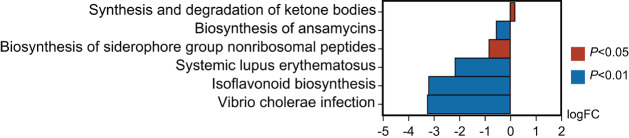
Figure6 **The KEGG differential pathway enrichment analysis**The abscissa is the logarithmic conversion of metabolic pathway abundance in cancer tissues relative to that in adjacent normal tissues. The positive values indicate enrichment of the pathway in cancer tissues, while the negative values indicate enrichment in adjacent normal tissues. The ordinate shows the different metabolic pathways. Different colors designate the P value annotations. LogFC, log2 (fold change).

To further explore the potential functional pathways based on the microbial community composition, we used PICRUSt2 software to predict the functional composition of the sample by the abundance of the marker gene sequence. The KEGG enrichment analysis results showed that the pathway of ketone body synthesis and degradation was enriched in cancer tissues ( *P*< 0.05), while pathways enriched in the adjacent normal tissues mainly involves the metabolism of terpenoids and polyketides, as well as immune and infectious diseases ( Figure 6). These results suggested that dominant taxa in cancer tissues may promote tumorigenesis through ketone body-related metabolic pathways.

## Discussion

In the past, the stomach was considered unsuitable for microbial growth due to its acidic environment, but the discovery of *H*. *pylori* changed that perception. With the rapid development of molecular technologies such as high-throughput sequencing, researchers have discovered a richer microbial community in the stomach, whose composition is influenced by factors such as *H*. *pylori*, diet, age, disease states, drug use, surgical interventions and inflammation [23]. Subsequently, *H*. *pylori* was shown to be the most important member of the gastric microbiome which is associated with many gastrointestinal diseases, such as peptic ulcer, gastric noncardia adenocarcinoma, and gastric mucosa-associated lymphoid tissue lymphoma [24]. The abundance of *H*. *pylori* can affect the composition of the gastric microbiota and thus promote the development of disease, and *H*. *pylori*-negative individuals have a higher diversity of microbiota [25]. Studies have revealed that, when *H*. *pylori* is positive, at the phylum level, the abundance of *Proteobacteria* in cancer tissues is slightly increased and the overrepresentation of *Actinobacteria* is decreased. Furthermore, *Campylobacterales* becomes the most abundant taxon at the order level [26]. However, the colonization of *H*. *pylori* decreases or even disappears in the late stage of carcinogenesis, and the eradication of *H*. *pylori* cannot completely prevent the development of gastric cancer, indicating that factors other than *H*. *pylori* promote the development of gastric cancer [27].

To investigate the effect of NHPHs on the local tumor microenvironment, we compared the microbiome of cancer tissues and adjacent normal tissues in the same individual and found significant differences in microbial composition between the two groups. All 10 patients in this study were negative for *H*. *pylori* infection. Compared with that in the adjacent normal tissues, the richness and genetic diversity of the microbial community in cancer tissues were significantly reduced. The dominant bacterial phyla were *Proteobacteria*, *Thermi*, *Actinobacteria*, *Bacteroidetes* and *Firmicutes* in the two groups, which is consistent with that of healthy controls [28]. The differences in the microbial community composition between the two groups were mainly reflected at the genus level and below. The relatively enriched genera in cancer tissues reported previously mainly include *Lactobacillus*, *Prevotella* and *Fusobacterium*, most of which belong to the oral flora [ 29, 30]. In this study, we found that other genera, such as *Cupriavidus*, *Sphingomonas*, *Brevundimonas*, *Caulobacter*, *Herbaspirillum*and *Acidovorax* are enriched. Of these, *Sphingomonas* has been reported to be associated with gastric disease and elevated abundance of *Sphingomonas* was found both in chronic gastritis patients and in gastric cancer patients without lymph node metastasis [31]. The remaining taxa have not been reported in gastric cancer but have been found in other tumors, such as bladder cancer, colorectal cancer, lung cancer and breast cancer [ 32– 36]. For example, *Cupriavidus* was found to be significantly abundant in patients with nonmuscle invasive bladder cancer [37]. In colorectal cancer, decreased abundance of *Brevundimonas* may contribute to the development of cancer, while higher abundance of *Herbaspirillum* is associated with NRAS mutation [ 38, 39]. *Acidovorax* exhibits higher abundance in lung squamous cell carcinoma with TP53 mutation but not in adenocarcinoma [40]. Among the taxa enriched in adjacent normal tissues in our study, the LDA value of *Ochrobactrum* was the highest. Previous studies have shown that *Ochrobactrum* is significantly enriched in patients with early gastric cancer compared to patients with chronic gastritis [41]. However, the results of our study showed that *Ochrobactrum* was more abundant in adjacent normal tissues than in cancer tissues.

Some researchers suggested that tumors are a metabolic disease and that cancer cells have different metabolic characteristics than normal cells [42]. More and more evidence supports that microorganisms can interact with the host through metabolites and play an important role in the development of diseases. Ketone bodies play important roles in mammalian physiopathology, such as inflammation, oxidative stress and immune response, and can affect cell proliferation by regulating energy metabolism [ 43, 44]. In this study we performed functional enrichment analyses on the microbial community, and found that ketone body-related metabolic pathways are relatively enriched in cancer tissues, indicating that related taxa have a metabolic regulation effect on ketone bodies in the tumor microenvironment. However, the metabolism of ketone bodies is dynamic and corresponds to the nutritional conditions of the body. Therefore, it is necessary to explore the functional changes of microorganisms in cancer tissues to better understand their potential role in the development of gastric cancer.

This study has obvious advantages. We used a uniform operating process and a mature analysis platform to ensure the reliability of the results. However, the study also has certain limitations. The sample size of this study is relatively small, which may introduce potential bias into the results. In addition, we did not further explore the mechanism by which the dominant taxa influence the metabolic pathways associated with ketone bodies. The above important issues will be investigated in future work.

In summary, in this study we demonstrated the differences in the composition of the mucosa-related microbiome between cancer tissues and adjacent normal tissues in patients with gastric cancer who were *H*. *pylori* negative, and predicted the role of related metabolic pathways in the tumor local microenvironment. Our data provide theoretical value for the study of molecular biomarkers and potential therapeutic interventions for gastric cancer.

## Supporting information

227TableS1

227TableS3

227TableS2
