# Supplementary material for: The composition and functional profile of the microbial communities in human gastric cancer tissues and adjacent normal tissues: Microbial communities in gastric cancer
Source: Acta Biochim Biophys Sin (Shanghai). 2021 Dec 31;54(1):47–54. doi: 10.3724/abbs.2021010 (PMC9909298; doi:10.3724/abbs.2021010)
Supplement: 227TableS1 [file 227TableS1.docx]

**Table S1.** **Sequence information for each sample**

| Sample ID (i) | Input  (Seqs) | Filtered  (Seqs) | Denoised  (Seqs) | Merged  (Seqs) | Nonchimeric  (Seqs) | Nonsingleton(Seqs) |
| --- | --- | --- | --- | --- | --- | --- |
| T1 | 33794 | 33518 | 33200 | 32097 | 28628 | 28624 |
| T2 | 32244 | 31899 | 31634 | 30972 | 30179 | 30177 |
| T3 | 39928 | 39593 | 39282 | 38580 | 35725 | 35716 |
| T4 | 33349 | 32964 | 32527 | 31733 | 31539 | 31508 |
| T5 | 31985 | 31619 | 31384 | 30757 | 30190 | 30181 |
| T6 | 29534 | 29281 | 28995 | 28202 | 25562 | 25559 |
| T7 | 40324 | 39981 | 39715 | 38917 | 38334 | 38321 |
| T8 | 32680 | 32437 | 32134 | 31369 | 28319 | 28318 |
| T9 | 47283 | 46885 | 46631 | 45782 | 44483 | 44469 |
| T10 | 38313 | 38028 | 37679 | 36880 | 33073 | 33067 |
| N1 | 47680 | 47606 | 46454 | 43295 | 40200 | 39702 |
| N2 | 51953 | 51864 | 50513 | 47708 | 44073 | 43614 |
| N3 | 51200 | 51106 | 50158 | 47741 | 44896 | 44457 |
| N4 | 48555 | 48449 | 47408 | 45086 | 42306 | 41965 |
| N5 | 50209 | 50094 | 49027 | 46380 | 44081 | 43588 |
| N6 | 48054 | 47949 | 46951 | 44586 | 41189 | 40692 |
| N7 | 34273 | 33998 | 33758 | 33327 | 33084 | 33073 |
| N8 | 49396 | 49292 | 48173 | 45271 | 42582 | 42219 |
| N9 | 48335 | 48206 | 47056 | 43966 | 42211 | 41810 |
| N10 | 36118 | 35723 | 35461 | 35008 | 34873 | 34866 |

i, order number for each sample. Seqs, sequences of the sample. T, cancer tissues. N, adjacent normal tissues.
