# Supplementary material for: The composition and functional profile of the microbial communities in human gastric cancer tissues and adjacent normal tissues: Microbial communities in gastric cancer
Source: Acta Biochim Biophys Sin (Shanghai). 2021 Dec 31;54(1):47–54. doi: 10.3724/abbs.2021010 (PMC9909298; doi:10.3724/abbs.2021010)
Supplement: 227TableS3 [file 227TableS3.docx]

**Table S3.** **The relative abundance of each taxon** **(top 10)** **at the genus level.**

| Taxa | T | N |
| --- | --- | --- |
| *Acinetobacter* | 0.3102 | 0.1699 |
| *Cupriavidus* | 0.2740 | 0.0709 |
| *Sphingomonas* | 0.1256 | 0.0233 |
| *Thermus* | 0.0591 | 0.0731 |
| *Ochrobactrum* | 0.0106 | 0.1038 |
| *Pseudomonadaceae_Pseudomonas* | 0.0780 | 0.0339 |
| *Pelomonas* | 0.0024 | 0.0673 |
| *Sediminibacterium* | 0.0014 | 0.0491 |
| *Sphingobium* | 0.0269 | 0.0124 |
| *Cryocola* | 0.0000 | 0.0387 |
| Others | 0.1118 | 0.3577 |

T, cancer tissues. N, adjacent normal tissues.
