# Supplementary material for: The composition and functional profile of the microbial communities in human gastric cancer tissues and adjacent normal tissues: Microbial communities in gastric cancer
Source: Acta Biochim Biophys Sin (Shanghai). 2021 Dec 31;54(1):47–54. doi: 10.3724/abbs.2021010 (PMC9909298; doi:10.3724/abbs.2021010)
Supplement: 227TableS2 [file 227TableS2.docx]

**Table S2.** **The relative abundance of each taxon** **(top 10)** **at the phylum level.**

| Taxa | T | N |
| --- | --- | --- |
| *Proteobacteria* | 0.9160 | 0.6810 |
| *Thermi* | 0.0592 | 0.0745 |
| *Actinobacteria* | 0.0094 | 0.0932 |
| *Bacteroidetes* | 0.0069 | 0.0784 |
| *Firmicutes* | 0.0049 | 0.0474 |
| *Cyanobacteria* | 0.0011 | 0.0115 |
| *Chloroflexi* | 0.0012 | 0.0012 |
| *Acidobacteria* | 0.0004 | 0.0018 |
| *Fusobacteria* | 0.0000 | 0.0018 |
| *Planctomycetes* | 0.0002 | 0.0015 |
| Others | 0.0008 | 0.0079 |

T, cancer tissues. N, adjacent normal tissues.
